# Sequence Conservation in *Plasmodium falciparum* α-Helical Coiled Coil Domains Proposed for Vaccine Development

**DOI:** 10.1371/journal.pone.0005419

**Published:** 2009-05-25

**Authors:** Caroline Kulangara, Andrey V. Kajava, Giampietro Corradin, Ingrid Felger

**Affiliations:** 1 Swiss Tropical Institute, Basel, Switzerland; 2 Centre de Recherches de Biochimie Macromoleculaire, FRE-2593 CNRS, Montpellier, France; 3 Department of Biochemistry, University of Lausanne, Epalinges, Switzerland; Queensland Institute of Medical Research, Australia

## Abstract

**Background:**

The availability of the *P. falciparum* genome has led to novel ways to identify potential vaccine candidates. A new approach for antigen discovery based on the bioinformatic selection of heptad repeat motifs corresponding to α-helical coiled coil structures yielded promising results. To elucidate the question about the relationship between the coiled coil motifs and their sequence conservation, we have assessed the extent of polymorphism in putative α-helical coiled coil domains in culture strains, in natural populations and in the single nucleotide polymorphism data available at PlasmoDB.

**Methodology/Principal Findings:**

14 α-helical coiled coil domains were selected based on preclinical experimental evaluation. They were tested by PCR amplification and sequencing of different *P. falciparum* culture strains and field isolates. We found that only 3 out of 14 α-helical coiled coils showed point mutations and/or length polymorphisms. Based on promising immunological results 5 of these peptides were selected for further analysis. Direct sequencing of field samples from Papua New Guinea and Tanzania showed that 3 out of these 5 peptides were completely conserved. An *in silico* analysis of polymorphism was performed for all 166 putative α-helical coiled coil domains originally identified in the *P. falciparum* genome. We found that 82% (137/166) of these peptides were conserved, and for one peptide only the detected SNPs decreased substantially the probability score for α-helical coiled coil formation. More SNPs were found in arrays of almost perfect tandem repeats. In summary, the coiled coil structure prediction was rarely modified by SNPs. The analysis revealed a number of peptides with strictly conserved α-helical coiled coil motifs.

**Conclusion/Significance:**

We conclude that the selection of α-helical coiled coil structural motifs is a valuable approach to identify potential vaccine targets showing a high degree of conservation.

## Introduction

The majority of known malaria antigens are highly polymorphic [1]. Tandem repeats are found in central domains of many antigens giving rise to extensive length polymorphism (LP) [2]. In addition, single nucleotide polymorphisms (SNPs) are abundant in antigenic genes, with 65% of SNPs on a genome-wide scale being non-synonymous (i.e the nucleotide substitution results in an amino acid change) [3]. The genetic diversity of new vaccine candidates is generally determined in the preclinical characterization of the candidate. High levels of polymorphism in malaria antigens are thought to be part of the parasite's strategy to avoid destruction by the host's immune defense. By including polymorphic sequences in a malaria vaccine, variant-specific immune responses will be elicited. As a consequence, alleles distinct form the vaccine molecule will be favored by selective advantage giving rise to escape variants. This situation was observed by molecular and immunological monitoring in the Phase I/IIb trial of the malaria vaccine Combination B that, in addition to two other components, contained almost the full length of merozoite surface protein 2 (MSP2) allele of the 3D7 cloned parasite line [4]. In vaccine recipients, a lower prevalence of the 3D7-type genotype was observed and genotypes belonging to the alternative allelic family were responsible for a higher incidence of malaria episodes [5]. A significant strain-specific humoral response was directed against the repetitive and family-specific MSP2 domains, whereas only low antibody titres were observed against conserved domains of MSP2 [6]. Similarly, a strain-specific response was observed in a challenge trial in Aotus monkeys with the two alleles of MSP1_42_ [7]. There are also contrasting findings from clinical trial of RTS,S where no selection was observed in break-through infections for SNPs in the circumsporozoite protein T-cell-epitope regions [8]. The question remains whether the inclusion of more than one allelic form of an antigen can compensate substantial polymorphism [9]. As for MSP2, the inclusion of two variants into a vaccine has been proposed for MSP3 [6], [10]. So far there is little experimental evidence that multi-allele vaccines actually reduce morbidity in contrast to single antigen vaccines [4]. An other interesting aspect in immune evasion is that naturally occurring variants of the same epitope can prevent memory T cells effector functions referred as “altered peptide ligand” antagonism [11], [12]. The above examples highlight a major obstacle for vaccine development posed by polymorphism in vaccine candidates. By using non-polymorphic domains of antigens, selection of vaccine escape variants could be avoided. A further important consideration in vaccine development is the complexity of candidate molecules in the vaccine formulation. If more variants are required in order to cover the major alleles found world-wide, highly complex mixtures, particularly for multi-component vaccines, would result; thus risking high costs and potential antagonistic effects [4].

Our approach to discover novel vaccine candidates is based on the selection of protein segments with defined structural motifs, with emphasis on identifying conserved domains of antigens. A genome-wide bioinformatic approach was taken to identify potential candidates that contain an α-helical coiled coil motif [13]. The α-helical coiled coils share a (**abcdefg**)_n_ motif containing hydrophobic residues at positions **a** and **d** and generally polar in the remaining positions. Chemically synthesized short peptides consisting of this motif can fold into their native structure. This is an appealing characteristic and represents a new approach to malaria vaccine development. The use of synthetic peptides over recombinantly expressed proteins in vaccines is advantageous because no expression or elaborate purification system is required, making the development process much less tedious and time consuming [14]. A further advantage of α-helical coiled coil motifs is that they are recognized by conformational dependent antibodies [15]. These coiled coil motifs are highly abundant in the eukaryotic cell. They are found in about 10% of all protein sequences [16]. This widespread occurrence in nature is explained by the broad range of function pertaining to the specific design of their coiled coil domains [17]. The crucial biological function of this domain has been investigated in numerous proteins. Generally, α-helical coiled coil domains serve as oligomerization motifs in proteins.

The rationale for our focus on peptides with little or no polymorphism was that these coiled coil regions were immunogenic in mice and well recognized by naturally occurring antibodies [13] (Olugbile et al., manuscript in preparation). In addition, affinity purified antibodies against these peptides killed parasites *in vitro* as shown by an assay involving antibody-dependent cellular inhibition [13], [18], [19]. Presence of hydrophobic residues in **a** and **d** positions is important for formation of the critical interhelical interactions while mostly hydrophilic residues in the remaining positions are exposed on the surface of the α-helical coiled coil motif and assumed to function as sites for protein interaction. Such structural and functional constraints associated with coiled coil domains likely signify these motifs are under purifying selection and led us to expect and investigate sequence conservation.

In an attempt to elucidate the relationship between coiled coil structure and sequence conservation, we analyzed the polymorphism in 166 synthetic peptides, previously identified in a genome-wide selection process [13]. Many of these molecules have undergone immunological testing [13] and some have successfully entered the vaccine development pipeline. 14 peptides included in the analysis were further assessed in 13 culture strains. The sequence diversity of 5 of these 14 peptides was also investigated in parasite populations from endemic countries.

## Materials and Methods

### Ethics Statement

Research clearance for blood sampling and genetic analysis of parasites was granted by the Tanzanian Commission for Science and Technology and by the Medical Research and Advisory Committee of the Ministry of Health in Papua New Guinea.

### Parasite culture

The culture strains were grown in 10 cm Petri dishes and cultured by standard methods in an atmosphere of 93% N_2_, 4% CO_2_, 3% O_2_ at 37°C as described previously [20]. The culture medium was RPMI 1640 10.44 g/L, supplemented with Hepes 5.94 g/L, Albumax II 5 g/L, hypoxanthine 50 mg/L, sodium bicarbonate 2.1 g/L and neomycin 100 mg/L.

### Polymorphism study in culture strains

Genetic diversity of 14 peptides spanning the α-helical coiled coil region of 10 hypothetical proteins was assessed in 13 *in vitro* culture strains (3D7, W2mef, HB3, ITG2F6, IFA18, FVO, 7G8, K1, RO33, MAD20, FCR3, RFCR3 and FC27). The geographical origin of each strain is listed in **Table 1**. Genomic DNA was isolated with phenol/chloroform extraction. PCR primers used to amplify the α-helical coiled-coil region are listed in **Table S1**. 63 blood samples derived from 1–5 year old children from Ifakara, Tanzania with uncomplicated acute malaria. These samples were collected in the course of an antimalarial drug trial [21]. 19 samples were asymptomatic community samples from Mugil village, Papua New Guinea [22]. Genomic DNA was isolated with phenol/chloroform extraction or the QIAamp DNA Blood Mini Kit 250 (Qiagen). PCR conditions consisted of denaturation at 94°C for 5 min followed by 35 cycles of denaturation (94°C for 1 min), annealing (50°C for 1 min) and extension (72°C for 1 min). The reaction products were incubated at 72°C for 10 min to ensure complete DNA extension. The PCR products were directly sequenced and aligned using Auto Assembler software to screen for SNPs and LP within the sequences corresponding to the peptides.

**Table 1:** Origin of culture strains.

culture strain	origin
3D7	Airport malaria (Amsterdam)
FC27	Madang, PNG
MAD20	PNG
RFCR3	Gambia
FCR3	Gambia
W2mef	Indochina
K1	Thailand
Hb3	Honduras
IFA18	Ifakara, Tanzania
RO-33	Ghana
ITG2F6	Brazil
FVO	Vietnam
7G8	Brazil

### RNA isolation and cDNA synthesis

Due to large introns in the genomic DNA corresponding to P38 and P77 cDNA was produced for sequencing of the 13 culture strains (3D7, W2mef, HB3, ITG2F6, IFA18, FVO, 7G8, K1, RO33, MAD20, FCR3, RFCR3 and FC27). 10 ml of parasite culture of 5% mixed stage parasites were lysed in 3 ml of Trizol (Invitrogen) and RNA was extracted with 0.2 volumes of chloroform and precipitated with 0.8 volumes of isopropanol. The extraction was repeated in half of the original Trizol volume to reduce contamination with gDNA. Residual gDNA was digested twice with RQ1 Dnase (Promega) according to the manufacturer's protocol in a total volume of 50 µl. RNA was dissolved in 25 µl of 5 mM Tris/0.5 mM EDTA and 9.5 µl RNA was used for the reverse transcription by AffinityScript Multiple Temperature Reverse Transcriptase (Stratagene) with random primers (Invitrogen) as described by the manufacturer. P38 and P77 sequences were amplified from cDNA with Advantage cDNA polymerase (BD Biosciences) using the primers listed in **Table S1**. To determine gDNA contamination the corresponding peptide sequences were amplified with Advantage Taq from RNA processed simultaneously without the addition of reverse transcriptase.

### Identification of α-helical coiled coil motif

For the original genome-wide selection of coiled coil domains, we generated 25 residues-long<-helical coiled coil profiles [13] by using *pftools* package [23]. This profile was constructed by using a multiple alignment of amino acid sequences corresponding to the known α-helical coiled coil domains found in the Protein Data Bank (PDB) [24], release 2006. In this work, the profile was updated by adding new sequences of the known coiled coil structures from PDB (release 2008). The score of this profile reflects the level of similarity of an analyzed amino acid sequence to the typical coiled coil motif which was deduced from the alignment of the known coiled coils. Tests of this profile against a sequence database of proteins with the known 3D structures showed that (1) the scores above 3.0 corresponded exclusively to coiled coil structures; (2) some coiled coil structures may have scores above 2.1. The 2.1 cut-off level was chosen for the first stage of the identification procedure to include most of the putative coiled coils. Subsequently, the selected coiled coil regions were tested manually for the presence of the characteristic heptad repeats. Although all putative α-helical coiled coil domains identified in the *P. falciparum* genome share the heptad repeat sequence motif, they can be distinguished by fidelity of the heptad repetitions. We subdivided the analyzed coiled coil regions into two groups. The first group contains peptides with perfect, or, in case of one or more SNPs, almost perfect tandem repetition of a certain sequence motif. The length of the perfect repeat either coincides with the length of the 7 residue coiled coil repeat or is divisible by 7. The second group of imperfect repeats is characterized by the repetition of amino acid residues with similar physico-chemical properties rather than by repeat units of exactly the same amino acid residues. Both types of repeats contain hydrophobic residues at positions **a** and **d** of the heptads and polar residues in the remaining positions. In this work, we assed the extend of polymorphism in the identified α-helical coiled coil domains and examined the polymorphism in perfect or almost perfect repeats as opposed to that in imperfect repeats.

## Results

The extensive genetic diversity of blood stage antigens is one of the key challenges in vaccine development against malaria. After the selection of 166 novel blood stage vaccine candidates, all harbouring α-helical coiled coil motif, we undertook a comprehensive *in silico* analysis of these domains. In addition, we performed an in depth molecular epidemiological analysis on selected peptides that proved to be the most promising vaccine candidates according to an immunological evaluation process [13].

SNP data for a maximum of 15 *P. falciparum* culture strains are currently available in the PlasmoDB 5.4 database (http://PlasmoDB.org), the official database of the *P. falciparum* genome sequencing consortium. Unfortunately, PlasmoDB 5.4 does not incorporate information on insertions or deletions. Nevertheless, insertions and deletions are thought to provide as much diversity as SNPs in *P. falciparum* [25]. In order to determine the full extent of diversity in recently identified vaccine candidates [13], we have analyzed the polymorphism of the selected α-helical coiled coil regions in 13 different culture strains and in cross sectional field samples from malaria endemic regions. Direct sequencing made it possible to detect both, SNP and LP.

### Genetic Diversity in *in vitro* culture strains of *P. falciparum*

**Figure 1:**
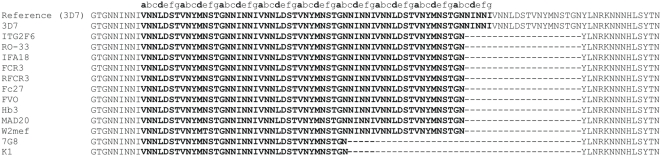
Alignment of P1 sequences amplified from 13 culture strains. Two types of deletions were detected in the PFA0170c gene: a longer deletion (del1) between T1599 and T1726 and a shorter deletion (del2) T1662 and T1726. On the protein level del1 results in the loss of NINNIVNNLDSTVNYMNSTGNNINNIVNNLDSTVNYMNSTGN amino acid motif between N533 and Y576 and del1 in the loss of NINNIVNNLDSTVNYMNSTGN between N554 and Y576 of P1. The peptide sequences P1 is indicated with bold letters. The heptad reapets are marked on top of the alignment sequence as a repeated abcdefg motif in which the hydrophobic position a (bold) and d (bold) are highlighted.

**Figure 2:**
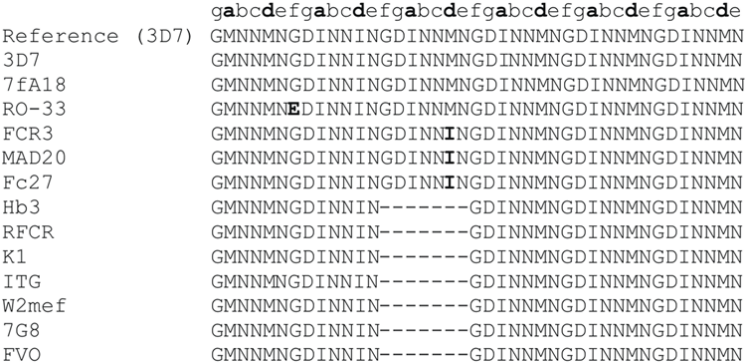
Alignment pf P14 sequences amplified from 13 culture strains. A deletion of the GDINNMN amino acid motif was observed with a frequency of 0.54 (7/13). In addition an imperfect repeat (GDINNIN) of this motif was detected with a frequency of 0.23 (3/13).

**Figure 3:**
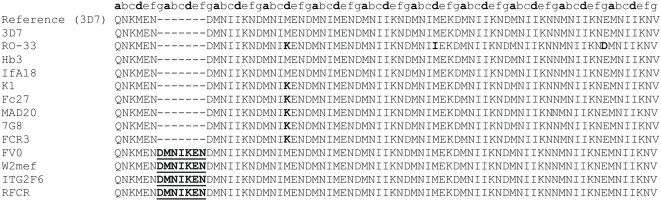
Alignment of P83 sequences amplified from 13 culture strains. SNP in P83 at nt position 863 T→A results in an amino acid change from Methionine (M) to a Lysine (K) at position 288 in PFC0345w gene product (red). This SNP was observed with a frequency of 0.38 (5/13). An insertion of DMNIKEN amino acid motif (bold and underlined) was detected with a frequency of 0.31 (4/13).

**Table 1** lists the 13 strains that were analyzed for SNPs and LP by PCR and direct sequencing of the corresponding 14 most promising peptides selected from the preclinical evaluations. Primers used for PCR amplification and sequencing are listed in **Table S1**. Polymorphism results are presented in **Table 2**. In addition we have included all information on polymorphism that is publicly available at PlasmoDB 5.4. Peptides P1, P14 and P83 showed either LP alone (P1, **Figure 1**) or both types of polymorphism, SNP plus LP (P14, **Figure 2** and P83 **Figure 3**). Peptides showing LP revealed tandem repeats and differed from each other by 1 to 3 heptad repeat units. Therefore, these mutations do not introduce a frame shift into the coiled coil motif. For example, P83 corresponding to the α-helical coiled coil domain of the gene product of PFC0345w shows a duplication of the heptamer DMNIKEN between amino acids N276 and D277, and was detected with a frequency of 0.31 (4/13) in 4 culture strains (**Table 2**).

**Table 2:** Genetic diversity in the 14 peptides sequences.

peptide^a^	Gene ID PlasmoDB 5.4	SNP data extracted from PlasmoDB 5.4			SNPs detected by sequencing of culture strains^b^		
		position	type of mutation^c-f^	frequency	position	type of mutation^c-f^	frequency
P1 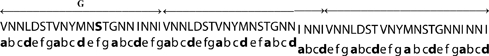	PFA0170c	nt 1525 A→T nt 1518 A→G nt 1581 G→A	ns (S509G) s s	1/15 1/15 1/15	nt 1599 nt 1662	Del Del	2/13 10/13
P2 TISSLSNKIVNYESKIEELEKELKEVK g**a**bc**d**efg**a**bc**d**efg**a**bc**d**efg**a**bc**d**e	PFB0145c	conserved			conserved		
P5 IIDIKKHLEKLKIEIKEKKEDLENL **a**bc**d**efg**a**bc**d**efg**a**bc**d**efg**a**bc**d**		conserved			conserved		
P8 IKTMNTQISTLKNDVHLLNEQDKLNNEKGTLNSKISEL **a**bc**d**efg**a**bc**d**efg**a**bc**d**efg**a**bc**d**efg**a**bc**d**ef**a**bc**d** NVQIMDL efg**a**bc**d**		conserved			conserved		
P9 LLSKDKEIEEKNKKIKELNNDIKKL **a**bc**d**efg**a**bc**d**efg**a**bc**d**efg**a**bc**d**		conserved			conserved		
P13 LDENEDNIKKMKSKIDDMEKEIKYR **a**bc**d**efg**a**bc**d**efg**a**bc**d**efg**a**bc**d**		conserved			conserved		
P14 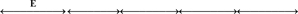 GMNNMN**G**DINNINGDINNMNGDINNMNGDINNMNGDIN g**a**bc**d**efg**a**bc**d**efg**a**bc**d**efg**a**bc**d**efg**a**bc**d**efg**a**b NMN c**d**e	PFC0245c	nt 6662 C→T nt 6726 T→A	ns (G2221E) s	1/10 1/8	nt 2227	Del (GDINNMN)	7/13
P27 KKRNVEEELHSLRKNYNIINEEIEEIT g**a**bc**d**efg**a**bc**d**efg**a**bc**d**efg**a**bc**d**e	PFF0165c	conserved			conserved		
P35 ARDDIQKDINKMESELINVSNEINRLD g**a**bc**d**efg**a**bc**d**efg**a**bc**d**efg**a**bc**d**e	PF14_0045	conserved			conserved		
P38 NITNINKNIENIKNDMSNLNNMNDSNQ g**a**bc**d**efg**a**bc**d**efg**a**bc**d**efg**a**bc**d**e	PF14_0089	conserved			conserved		
P77 EKLKKYNNEISSLKKELDILNEKMGKCT fg**a**bc**d**efg**a**bc**d**efg**a**bc**d**efg**a**bc**d**e	PF08_0048	conserved			conserved		
P79 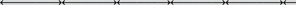 NEMNKEVNKMNEEVNKMNEEVNKMNEEVNKMNKEVNKM bc**d**efg**a**bc**d**efg**a**bc**d**efg**a**bc**d**efg**a**bc**d**ef**a**bc**d**e 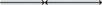 DEEVNKMNKEVNKMNK fg**a**bc**d**efg**a**bc**d**efg	PFB0315w	nt 4845 G→A	s	1/12	conserved		
P83 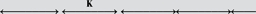 QNKMENDMNIIKNDMNI**M**ENDMNIMENDMNIIKNDMNI **a**bc**d**efg**a**bc**d**efg**a**bc**d**efg**a**bc**d**efg**a**bc**d**ef**a**bc**d** 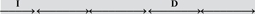 **M**EKDMNIIKNDMNIIKNNMNIIKN**E**MNIIKNV **a**bc**d**efg**a**bc**d**efg**a**bc**d**efg**a**bc**d**efg**a**bc**d**	PFC0345w	nt 863 T→A nt 927 G→A nt 999 A→C	ns (M288K) ns (M309I) ns (E333D)	4/9 1/10 1/10	nt 863 T→A nt 927 G→A nt 999 A→C nt 827	ns (M288K) ns (M309I) ns (E333D) dup (DMNIKEN)	5/13 1/13 1/13 4/13
P90 TKKLNKELSEGNKELEKLEKNIKELEETNNTLENDIKV g**a**bc**d**efg**a**bc**d**efg**a**bc**d**efg**a**bc**d**efg**a**bc**d**efg**a**b	PFD0520c	nt 195 A→G	s	1/9	conserved		

a Peptide sequences are aligned with the coiled coil heptad repeat (abcdefg); lines with arrows over some sequences indicate tandem repeats.

b *P. falciparum* in vitro cultre strains 3D7, FC27, K1, IFA18, FV0, 7G8, HB3, ITG2F6, RO-33, W2mef, FCR3S1.2, RFCR3, MAD20

c non synonymous (ns);

d synonymous (s);

e deletion (del);

f duplication (dup).

Some SNPs were prevalent in our culture strains. For P83 the SNP at nt 863 T→A occurred with a frequency of 0.38 (5/13). This SNP was also described in PlasmoDB 5.4 and was observed with a frequency of 0.43 (6/14). Two additional SNPs were found for P83. A G→A substitution at nt position 927 resulted in a non-synonymous change from methionine (M) to isoleucine (I) at amino acid 309. This SNP was only found once and only in our culture strains. An additional SNP at nt position 999, arising from an A→C conversion, results in an amino acid change from glutamic acid (E) to aspartic acid (D), and was also found only once. These SNPs were also reported in PlasmoDB. With respect to the underlying heptad repeat positions labeled **a** *to* **g**, all SNPs detected in P83 were at hydrophobic positions **a** and **d**, however, these SNPs do not affect the coiled coil formation significantly and the antibody epitopes on the surface of the coiled coil likely remain conserved. It is worth mentioning that, in principle, one point mutation can change the oligomerization state of the coiled coil. However, the general rules governing the stoichiometry of the coiled coil structures are still largely unknown. Due to the fact that most of the observed non-synonymous SNPs represent change to the residues with similar physico-chemical properties (e.g. hydrophobic to hydrophobic), we assume that the oligomerization states of the coiled coils also remain conserved. One SNP of P83 leads to a change of a hydrophobic methionine (M288) to a charged lysine (K) in **d**-position of the heptamer unit underlying coiled coil structural motifs (**Table 2**). The charged residue in **d**-position favors a coiled coil dimer. However, we do not know the oligomerization state of the original P83 coiled coil, thus it is impossible to conclude whether the M288K mutant indeed changes the oligomerization state. Further experimental studies are required to clarify the oligomerization state of P83 wildtype and mutant.

### Diversity in parasite populations

**Figure 4:**
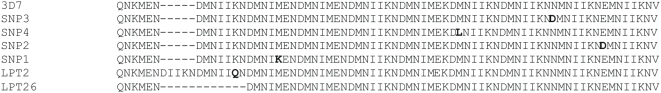
SNPs and LP of P83 sequences amplified in field samples from PNG and Tanzania. In PNG samples essentially only one SNP (SNP1) at nt position 863 T→A results in an amino acid change from Methionine (M) to a Lysine (K) at position 288 in PFC0345w gene product. This SNP was detected with a high frequency of 0.5 (4/8) and was also observed in culture strains. In Tanzanian samples 3 SNPs (SNP2, SNP3, SNP4; bolded) and 2 LP (LPT2, LPT26; insertion indicated in bold) occurring with low frequencies of 0.04 (1/27) were detected.

Five vaccine candidates, prioritized according to their performance in immunological and functional assays, were further analyzed in a small-scale molecular epidemiological survey. The basis for selection and results of the preclinical evaluation process were published previously [13], [14]. The five candidates were peptides P8, P27, P77, P83 and P90. In addition to showing promising results, all were conserved or showed limited polymorphism in culture strains (**Table 2**). The extent of sequence conservation was determined in field samples from two malaria endemic areas: Tanzania (TZ) and Papua New Guinea (PNG) (**Table 3**). P27 and P77 were found to be completely conserved. P90 is also conserved on the amino acid level in 23 samples from TZ and 31 samples from PNG, with only a synonymous SNP 195 A→G in samples from TZ with a frequency of 0.17 (4/23). This SNP was also reported in PlasmoDB 5.4 to occur in the GHANA1 strain (**Table 2**). The P8 sequence was conserved in culture strains, but in the field samples 2 SNPs were detected. 4 SNPs (SNP1-4) and 2 LP (LPT2, LPT26) were detected for P83 in both populations examined (**Figure 4**). Thus, in field samples 3/5 peptides showed complete conservation on the amino acid level and only minor polymorphism was observed for the remaining 2 candidates. This is in line with sequence diversity detected in culture strains where 3/14 peptides had shown LP or SNP plus LP.

**Table 3:** Genetic diversity of P27, P90, P83, P8 and P77 in *P. falciparum* positive field samples from Tanzania and Papua New Guinea.

peptide	Gene ID	SNPs detected by sequencing of field samples		
		nt position	frequency	type of mutation^a^^–^ [Table-fn nt108] [Table-fn nt109] d
P27	PFF0165c	conserved 46/46 (TZ)		
		conserved 17/17 (PNG)		
P90	PFD0520c	nt 195 T→C	4/23 (PNG)	s
			conserved 31/31 (TZ)	
P77	PF08_0048	conserved 14/14 (TZ)		
		conserved 10/10 (PNG)		
P8	PFB0145c	nt 1817 C→T	1/33 (TZ)	ns (T606I)
		nt 1856 T→A	1/29 (PNG)	ns (M619K)
P83	PFC0345w	nt 863 T→A	4/8 (PNG)	ns (M288K)
		nt 999 A→C	1/27 (TZ)	ns (E333D)
		nt 976 A→G	1/27 (TZ)	ns (N326D)
		nt 937 A→C	1/27 (TZ)	ns (M313L)
		nt 843 A→C	1/27 (TZ)	ns (K282Q)
		nt 844 A→C	1/27 (TZ)	ns (K282Q)
		nt 823	1/27 (TZ)	dup (DIIKN)
		nt 885	1/27 (TZ)	del (DMNIIKN)

a non synonymous (ns).

b synonymous (s).

c deletion (del).

d duplication (dup).

### Effects of polymorphism on the probability of α-helical coiled coil structure formation

A comprehensive *in silico* analysis was performed on 166 selected coiled coil sequences to determine the effects of SNP and LP on the probability to form an α-helical coiled coil. Our approach allowed the prediction of structure modifications caused by the known SNPs within our peptides, which are recorded in the SNP database in PlasmoDB

Overall we detected a high degree of sequence conservation in 166 predicted α-helical coiled coil domains. Only 29/166 peptides showed limited polymorphism. In one of the 29 polymorphic peptides the score fell below the cut-off (altered score bolded in **Table 4**). In contrast to the above result on P83 that had shown SNPs exclusively at hydrophobic residues, the majority of the SNPs in the 166 peptides were found at the surface positions **b**, **c**, **e**, **f** and **g** within the heptad repeat and are unlikely to destabilize the coiled coil structures.

**Table 4:** SNPs detected in peptide sequences based on the SNP data published in PlasmoDB 5.4

Peptide	Protein	Sequence	Frequency	Score a
P1	PFA0170c	3D7:		
		VNNLDSTVNYMN**S**TGNNINNIVNNLDSTVNYMNSTGNNINNIVNNLDSTVNYMNSTGNNINNI		2.570
		Hb3:		
		VNNLDSTVNYMN**G**TGNNINNIVNNLDSTVNYMNSTGNNINNIVNNLDSTVNYMNSTGNNINNI	1/15	2.570
P14	PFC0245c	3D7:		
		GMNNMN**G**DINNINGDINNMNGDINNMNGDINNMNGDINNMN		3.100
		RO-33:		
		GMNNMN**E**DINNINGDINNMNGDINNMNGDINNMNGDINNMN	1/10	3.450
P17	PFD0110w	3D7:		
		EKKLDILK**V**NISNINNSLDKLK		manual^b^
		GHANA1:		
		EKKLDILK**A**NISNINNSLDKLK	1/11	manual^b^
P23		3D7:		
		FQK**V**KEKAE**I**QKENIEKIKQEINTL		manual^b^
		7G8;FCB;K1:		
		FQKVKEKAE**N**QKENIEKIKQEINTL	4/10	manual^b^
		GHANA1:		
		FQK**I**KEKAE**N**QKENIEKIKQEINTL	1/10	manual^b^
P33	PF13_0277	3D7:		
		YIDDV**D**RDVENYDKGIANVDHHLNDVH		3.020
		106_1;D6;Dd2;FCC-2;FCR3;GHANA1;Hb3;IT;K1;RO-33		
		YIDDV**N**RDVENYDKGIANVDHHLNDVH	10/12	3.080
P34	MAL13P1.336	3D7:		
		NMNNMNNNMNNMNNNMNNNMNNMN**N**MN		3.570
		D6:		
		NMNNMNNNMNNMNNNMNNNMNNMN**S**MN	1/5	3.550
P36	PF14_0093	3D7:		
		SSNNLSDQINILNNNIQHINS**T**FNNLR		2.920
		Senegal3404:		
		SSNNLSDQINILNNNIQHINS**I**FNNLR	1/8	2.730
P45	PF11_0207	3D7:		
		EE**I**KEEIKEVKEEIKEVKEEIKEVKEEIKEVKEEIKE		4.550
		GHANA1:		
		EE**V**KEEIKEVKEEIKEVKEEIKEVKEEIKEVKEEIKE	1/2	4.550
P47	PFA0635c	3D7:		
		NHDTRINDYNKRLTEYNKRLT**E**YNKRLTEYTKRLNE		3.520
		Hb3:		
		NHDTRINDYNKRLTEYNKRLT**A**YNKRLTEYTKRLNE	1/9	3.320
P50	PFL1605w	3D7:		
		KNDINVQLDDINVQLDDIN**V**QLDDIN**I**QLDEINLN		3.290
		7G8:		
		KNDINVQLDDINVQLDDIN**I**QLDDINIQLDEINLN	1/10	3.270
		D10:		
		KNDINVQLDDINVQLDDINVQLDDIN**V**QLDEINLN	1/13	3.310
P51	PFD0985w	3D7:		
		DNNVNN**M**DNNVNNVDNNVNNVDNNLNNVDNNVNN		3.950
		Dd2:		
		DNNVNN**V**DNNVNNVDNNVNNVDNNLNNVDNNVNN	1/11	3.900
P54	MAL6P1.147	3D7:		
		**DS**MNNHKDDMNNYNDNINNYVESMNNYDDIMNK		2.470
		FCC-2:		
		D**N**MNNHKDDMNNYNDNINNYVESMNNYDDIMNK	1/9	2.470
		GHANA1:		
		**N**S**N**NNHKDDMNNYNDNINNYVESMNNYDDIMNK	1/9	**1.920**
P64	MAL6P1.131	3D7:		
		NNFVNNKMNNMNNMKNNMNNMNNIMNN**I**MN		3.100
		FCC-2:		
		NNFVNNKMNNMNNMKNNMNNMNNIMNN**N**MN	1/8	2.650
P81	PF07_0086	3D7:		
		NEMNKEVNKMNEEVNKMNEEVNKMN**E**EVNKM**NK**EVNKMDEEVNKMN		4.580
		D10;D6;GHANA1;Santa Lucia:		
		NEMNKEVNKMNEEVNKMNEEVNKMNEEVNKMN**E**EVNKMDEEVNKMN	4/9	4.580
		GHANA1:		
		NEMNKEVNKMNEEVNKMNEEVNKMN**K**EVNKM**D**KEVNKMDEEVNKMN	1/7	4.580
P83	PFC0345w	3D7:		
		QNKMENDMNIIKNDMNI**M**ENDMNIMENDMNIIKNDMNI**M**E		3.250
		KDMNIIKNDMNIIKNNMNIIKN**E**MNIIKNV		
		D10;FCC-2;V1_S:		
		QNKMENDMNIIKNDMNI**K**ENDMNIMENDMNIIKNDMNIME	4/9	3.220
		KDMNIIKNDMNIIKNNMNIIKNEMNIIKNV		
		RO-33:		
		QNKMENDMNIIKNDMNI**K**ENDMNIMENDMNIIKNDMNI**I**E	1/10	3.200
		KDMNIIKNDMNIIKNNMNIIKN**D**MNIIKNV		
P86	PFL1930w	3D7:		
		NFIKELELQIKNLNNEI**N**TLNDMLKDSEEEIRMLNHTLEEK		3.710
		7G8:		
		NFIKELELQIKNLNNEI**K**TLNDMLKDSEEEIRMLNHTLEEK	1/11	3.760
P94	PFD0970c	3D7:		
		ENINNMDEK**I**N**N**VDEQNNN**M**DEKINNVDEK**K**		3.460
		FCC-2:		
		ENINNMDEKINNVDEQNNNMDEKINNVDEK**I**	1/6	3.460
		D10;K1:		
		ENINNMDEK**K**N**K**VDEQNNNMDEKINNVDEKK	4/7	2.990
		D6;FCC-2:		
		ENINNMDEKIN**K**VDEQNNNMDEKINNVDEKK	3/5	2.970
		RO-33:		
		ENINNMDEKIN**K**VDEQNNN**I**DEKINNVDEKK	3/6	3.480
P97	PFL0650c	3D7:		
		DTANNVSEMQKIIHTFSLDIKDFKILFEALTKSIQLLNDN		3.390
		IENINKEIEL**L**KKKIST		
		FCC-2:		
		DTANNVSEMQKIIHTFSLDIKDFKILFEALTKSIQLLNDN	1/8	3.140
		IENINKEIEL**F**KKKIST		
P103	PFE0840c	3D7:		
		NNMNNININAYN**I**LKDINKYRNIVNNLDH		2.310
		Dd2;FCC-2		
		NNMNNININAYN**V**LKDINKYRNIVNNLDH	2/7	2.310
P116	PF13_0182	3D7:		
		RISNILFMLNNIKKEIENVKENILKYID**K**		2.570
		106_1;7G8;GHANA1;IT;Senegal3404;V1_S		
		RISNILFMLNNIKKEIENVKENILKYID**R**	6/11	2.570
P119	PF11_0240	3D7:		
		DNVEELNKDIE**N**LNNEIHEIEKMWLFIKK		3.010
		GHANA1:		
		DNVEELNKDIE**I**LNNEIHEIEKMWLFIKK	1/8	2.770
P122	PFI1475w	3D7:		
		Y**D**L**S**IYNKQLEEAHNLISVLEKRIDTLKK		2.930
		D10;Dd2;FCC-2		
		Y**N**LSIYNKQLEEAHNLISVLEKRIDTLKK	3/16	2.640
		7G8;D10;Dd2;FCC-2;K1;RO-33;V1_S		
		YDL**F**IYNKQLEEAHNLISVLEKRIDTLKK	7/16	2.640
P134	PF14_0535	3D7:		
		KQIDLNINNIDD**NK**NNIDDHINNIDD		2.750
		7G8:		
		KQIDLNINNIDD**HI**NNIDDHINNIDD	1/7	3.060
P144	PF13_0210	3D7:		
		HDVNNYQHDVNNYQQDVNNYQHDVNNYQHDVNNY**N**H		2.470
		Dd2;Hb3;RO-33;Senegal3403;V1_S		
		HDVNNYQHDVNNYQQDVNNYQHDVNNYQHDVNNY**H**H	4/5	2.470
P149	MAL13P1.252	3D7:		
		NLALNNLRNKLKLLQEDYNDLEKEYLYLKK**S**LSK		3.350
		Dd2:		
		NLALNNLRNKLKLLQEDYNDLEKEYLYLKK**G**LSK	1/8	3.350
P154	PFL1205c	3D7:		
		QFIILMVKLLSNVYEI**V**KKINSLNNNYTYLYIN		2.680
		7G8;D10;FCB;FCC2;GHANA1;Hb3;K1;Senegal3404		
		QFIILMVKLLSNVYEI**E**KKINSLNNNYTYLYIN	7/8	3.030
P156	PFD1030c	3D7:		
		KINSLKENIENMFDRIDNYIH**E**VISLKNFVNQ		2.820
		K1:		
		KINSLKENIENMFDRIDNYIH**V**VISLKNFVNQ	1/8	2.460
P160	PF11_0268	3D7:		
		MNISEFETKITELKEDINKINFMMK**D**FESKF		3.170
		7G8;D10;D6;Dd2;FCC-2;FCR3;GHANA1;Hb3;IT;K1;RO-33;Senegal3404;V1_S:		
		MNISEFETKITELKEDINKINFMMK**A**FESKF	13/13	3.020
P166	PFI1180w	3D7:		
		Y**Y**NNFDNYNNNFDNYNNNFDNYNNNFDNYN		2.420
		GHANA1:		
		Y**N**NNFDNYNNNFDNYNNNFDNYNNNFDNYN	1/9	2.420

a profile score for α-helical coiled coil formation;

b profile score was manually analyzed.

Positions of SNPs are indicated in bold.

For peptides P17 and P23 -wild type (in culture strain 3D7; **Table 4**), P17-mutant (in culture strain GHANA1), and P23-mutant (in culture strain 7G8, FCB, K1, GHANA1; **Table 4**) the length of the synthesized peptide was too short to be analyzed by the 25 residue-long profile. These short peptides were then analyzed manually and it was shown that mutations do not affect the heptad pattern and therefore do not prevent α-helical coiled coil formation.

## Discussion

One of the hurdles in vaccine development against erythrocytic stages of the parasite is the extensive polymorphism observed in blood stage antigens. A function of polymorphic epitopes may be to divert the effective response. In natural and artificially induced humoral responses, the polymorphic regions of antigens were found to be immunodominant [2], [10], [26], but it is not known whether polymorphic regions are better or worse than conserved regions as targets of protective immunity.

The fact that polymorphism is maintained in populations lead to the question whether this is due to immune selection through allele-specific protective responses. For several polymorphic antigens immune selection has been confirmed [10], [27]. Similarly, SNPs were demonstrated to be under balancing selection, and the frequency of SNPs as a signature of selection was used to identify new vaccine targets in known antigens [27] or in the entire *Plasmodium falciparum* genome [3].

Conserved regions were found to be less antigenic and immunogenic than polymorphic regions [6], [10], [26], [28]. To investigate whether conserved regions can elicit adequate protection, the effect of antibody responses to both the conserved and polymorphic regions of MSP3 was measured [10]. Antibodies against both regions were associated with a reduced risk to develop clinical malaria [10]. Moreover, antibodies against the conserved epitopes elicited in humans inhibited parasites growth *in vitro* as shown by the antibody-dependent cellular inhibition assay [29] and lead to rapid parasite clearance after injection to humanized mice [29], [30].

During preclinical evaluation of new vaccine candidates, both antigenicity and sequence conservation are generally determined. We have shown that the majority of our candidates were both conserved and antigenic. In sero-epidemiological surveys the most peptides were found to be recognized by sera of adults from malaria endemic countries [13] (own unpublished results). Immunogenicity of most of the peptides investigated in more detail was confirmed in mice or rabbits [13] (Olugbile, unpublished results). It remains to be shown whether our described strategy to select non-polymorphic epitopes for inclusion in a vaccine will lead to greater efficacy in a field trial.

We analyzed the degree of conservation in predicted α-helical coiled coil regions of all proteins expressed in the blood stages of the parasite. Sequencing revealed that SNPs observed in field samples did not seem to disturb the heptad motif and thus do not destabilize the coiled coil structure. SNPs mostly occurred at hydrophilic surface positions of the coil except for P83. It is likely that SNPs located at surface positions result in a decreased antibody response to the variant epitope and may lead to immune evasion. However, the extent of polymorphism detected in our candidates was very limited and thus might not create a major limitation for vaccine efficiency.

Our sequence analysis revealed that both SNPs and LP were preferentially observed in the α-helical coiled coil motifs containing almost perfect tandem repeats. The perfect repeat units either coincide with the 7-residue coiled coil repeat (e.g. P14, P45, P50, P51, P64, P81, P83, P144, P166) or covering two or more heptad repeats (e.g. P1, P94). This correlation between the repeats perfection and polymorphism leads to a practical recommendation for selection of vaccine candidates: when searching for α-helical coiled coil regions with a reduced level of polymorphism, one should avoid regions with almost perfect tandem repeats.

α-helical coiled coil domains were found to be crucial for the biological function of various proteins. Coiled coils have been shown to be involved in oligomerization, protein-protein interaction and complex formation. These features support many cellular processes such as membrane fusion, protein transport and cell motility [17]. But for the proteins investigated by it is not known whether the putative coiled coil domains are of any functional importance. If these domains play a role in protein function, purifying selection might counteract diversification and polymorphism. Sequence conservation due to functional constraints was reported from viral envelope proteins where the conserved region was found at those positions of the coiled coil that are responsible for protein-protein interaction [31].

Extensive polymorphism has been an issue for the most promising blood stage vaccine candidates, such as apical membrane antigen 1, MSP1 and 2 [6], [32]–[35]. In the past, vaccine research was focused on a limited number of vaccine candidates. Due to recent disappointing results from clinical trials where a number of vaccine candidates were not found to be immunogenic, safe, or protective against artificial challenge [36]–[38], new emphasis is laid upon the discovery of novel target antigens. If more of the current candidates fail, additional antigens are required for supplementing the vaccine pipeline. There is a great demand to identify new antigens that are both, immunogenic and conserved. It remains to be shown whether the strategy to include non-polymorphic antigens in a vaccine formulation will increase protection.

## Supporting Information

Table S1 (0.04 MB DOC)

## References

[1] Good MF (2005). Vaccine-induced immunity to malaria parasites and the need for novel strategies.. Trends Parasitol.

[2] Kemp DJ, Coppel RL, Anders RF (1987). Repetitive proteins and genes of malaria.. Annu Rev Microbiol.

[3] Mu J, Awadalla P, Duan J, McGee KM, Keebler J (2007). Genome-wide variation and identification of vaccine targets in the Plasmodium falciparum genome.. Nat Genet.

[4] Saul A, Fay MP (2007). Human immunity and the design of multi-component, single target vaccines.. PLoS ONE.

[5] Genton B, Betuela I, Felger I, Al-Yaman F, Anders RF (2002). A recombinant blood-stage malaria vaccine reduces Plasmodium falciparum density and exerts selective pressure on parasite populations in a phase 1-2b trial in Papua New Guinea.. J Infect Dis.

[6] Fluck C, Smith T, Beck HP, Irion A, Betuela I (2004). Strain-specific humoral response to a polymorphic malaria vaccine.. Infect Immun.

[7] Lyon JA, Angov E, Fay MP, Sullivan JS, Girourd AS (2008). Protection induced by Plasmodium falciparum MSP1(42) is strain-specific, antigen and adjuvant dependent, and correlates with antibody responses.. PLoS ONE.

[8] Enosse S, Dobano C, Quelhas D, Aponte JJ, Lievens M (2006). RTS,S/AS02A malaria vaccine does not induce parasite CSP T cell epitope selection and reduces multiplicity of infection.. PLoS Clin Trials.

[9] Saul A, Lawrence G, Smillie A, Rzepczyk CM, Reed C (1999). Human phase I vaccine trials of 3 recombinant asexual stage malaria antigens with Montanide ISA720 adjuvant.. Vaccine.

[10] Polley SD, Tetteh KK, Lloyd JM, Akpogheneta OJ, Greenwood BM (2007). Plasmodium falciparum merozoite surface protein 3 is a target of allele-specific immunity and alleles are maintained by natural selection.. J Infect Dis.

[11] Plebanski M, Lee EA, Hill AV (1997). Immune evasion in malaria: altered peptide ligands of the circumsporozoite protein.. Parasitology.

[12] Pouniotis DS, Proudfoot O, Minigo G, Hanley JL, Plebanski M (2004). Malaria parasite interactions with the human host.. J Postgrad Med.

[13] Villard V, Agak GW, Frank G, Jafarshad A, Servis C (2007). Rapid identification of malaria vaccine candidates based on alpha-helical coiled coil protein motif.. PLoS ONE.

[14] Corradin G, Villard V, Kajava AV (2007). Protein structure based strategies for antigen discovery and vaccine development against malaria and other pathogens.. Endocr Metab Immune Disord Drug Targets.

[15] Lu SM, Hodges RS (2002). A de novo designed template for generating conformation-specific antibodies that recognize alpha-helices in proteins.. J Biol Chem.

[16] Walshaw J, Woolfson DN (2001). Open-and-shut cases in coiled-coil assembly: alpha-sheets and alpha-cylinders.. Protein Sci.

[17] Burkhard P, Stetefeld J, Strelkov SV (2001). Coiled coils: a highly versatile protein folding motif.. Trends Cell Biol.

[18] Jafarshad A, Dziegiel MH, Lundquist R, Nielsen LK, Singh S (2007). A novel antibody-dependent cellular cytotoxicity mechanism involved in defense against malaria requires costimulation of monocytes FcgammaRII and FcgammaRIII.. J Immunol.

[19] Bouharoun-Tayoun H, Oeuvray C, Lunel F, Druilhe P (1995). Mechanisms underlying the monocyte-mediated antibody-dependent killing of Plasmodium falciparum asexual blood stages.. J Exp Med.

[20] Trager W, Jensen JB (1997). Continuous culture of Plasmodium falciparum: its impact on malaria research.. Int J Parasitol.

[21] Irion A, Felger I, Abdulla S, Smith T, Mull R (1998). Distinction of recrudescences from new infections by PCR-RFLP analysis in a comparative trial of CGP 56 697 and chloroquine in Tanzanian children.. Trop Med Int Health.

[22] Marfurt J, Mueller I, Sie A, Maku P, Goroti M (2007). Low efficacy of amodiaquine or chloroquine plus sulfadoxine-pyrimethamine against Plasmodium falciparum and P. vivax malaria in Papua New Guinea.. Am J Trop Med Hyg.

[23] Bucher P, Karplus K, Moeri N, Hofmann K (1996). A flexible motif search technique based on generalized profiles.. Comput Chem.

[24] Berman H, Henrick K, Nakamura H (2003). Announcing the worldwide Protein Data Bank.. Nat Struct Biol.

[25] Volkman SK, Sabeti PC, DeCaprio D, Neafsey DE, Schaffner SF (2007). A genome-wide map of diversity in Plasmodium falciparum.. Nat Genet.

[26] Franks S, Baton L, Tetteh K, Tongren E, Dewin D (2003). Genetic diversity and antigenic polymorphism in Plasmodium falciparum: extensive serological cross-reactivity between allelic variants of merozoite surface protein 2.. Infect Immun.

[27] Conway DJ, Cavanagh DR, Tanabe K, Roper C, Mikes ZS (2000). A principal target of human immunity to malaria identified by molecular population genetic and immunological analyses.. Nat Med.

[28] Osier FH, Polley SD, Mwangi T, Lowe B, Conway DJ (2007). Naturally acquired antibodies to polymorphic and conserved epitopes of Plasmodium falciparum merozoite surface protein 3.. Parasite Immunol.

[29] Druilhe P, Spertini F, Soesoe D, Corradin G, Mejia P (2005). A malaria vaccine that elicits in humans antibodies able to kill Plasmodium falciparum.. PLoS Med.

[30] Singh S, Soe S, Mejia JP, Roussilhon C, Theisen M (2004). Identification of a conserved region of Plasmodium falciparum MSP3 targeted by biologically active antibodies to improve vaccine design.. J Infect Dis.

[31] Lamb D, Schuttelkopf AW, van Aalten DM, Brighty DW (2008). Highly specific inhibition of leukaemia virus membrane fusion by interaction of peptide antagonists with a conserved region of the coiled coil of envelope.. Retrovirology.

[32] Bai T, Becker M, Gupta A, Strike P, Murphy VJ (2005). Structure of AMA1 from Plasmodium falciparum reveals a clustering of polymorphisms that surround a conserved hydrophobic pocket.. Proc Natl Acad Sci U S A.

[33] Crewther PE, Matthew ML, Flegg RH, Anders RF (1996). Protective immune responses to apical membrane antigen 1 of Plasmodium chabaudi involve recognition of strain-specific epitopes.. Infect Immun.

[34] Felger I, Irion A, Steiger S, Beck HP (1999). Genotypes of merozoite surface protein 2 of Plasmodium falciparum in Tanzania.. Trans R Soc Trop Med Hyg.

[35] Tetteh KK, Cavanagh DR, Corran P, Musonda R, McBride JS (2005). Extensive antigenic polymorphism within the repeat sequence of the Plasmodium falciparum merozoite surface protein 1 block 2 is incorporated in a minimal polyvalent immunogen.. Infect Immun.

[36] Vekemans J, Ballou WR (2008). Plasmodium falciparum malaria vaccines in development.. Expert Rev Vaccines.

[37] Genton B (2008). Malaria vaccines: a toy for travelers or a tool for eradication?. Expert Rev Vaccines.

[38] Bejon P, Mwacharo J, Kai OK, Todryk S, Keating S (2006). Immunogenicity of the candidate malaria vaccines FP9 and modified vaccinia virus Ankara encoding the pre-erythrocytic antigen ME-TRAP in 1–6 year old children in a malaria endemic area.. Vaccine.

