# Supplementary material for: Sequence Conservation in Plasmodium falciparum α-Helical Coiled Coil Domains Proposed for Vaccine Development
Source: PLoS One. 2009 May 25;4(5):e5419. doi: 10.1371/journal.pone.0005419 (PMC2683929; doi:10.1371/journal.pone.0005419)
Supplement: Table S1 — (0.04 MB DOC) [file pone.0005419.s001.doc]

**Table S1: Primer sequences used to amplify 14 peptides**

| Peptide | Gene ID | Forward primer | Reverse primer |
| --- | --- | --- | --- |
| P1 | PFA0170c | CTAAATTCGTATCTATTTCGTCAT | TTCAGTGCTAGTTATGTTCCA |
| P2 | PFB0145c | CTATAGAAATCGTTTCTCTTTCTT | AATAATTATAGCGATGAAATATCAA |
| P5 |  | CCATATGAAGGAAATTATGTTCTC | GGAAGAAACATTTCATAATATAG |
| P8 |  | CATCTCCCGTATCCTCACATCACAATC | ACGCACAAATAGAAAGTATAAGCATCGAA |
| P9 |  | GATCTACTTGTTGTGCATTACTTTG | CATTGAATGTTGTGATGATATAGA |
| P13 |  | AAGAGATGTTTCTTTATACGTAAGAG | AGATGAATTAAATGAAGAAGTGGAAA |
| P14 | PFC0245c | ATGATGTGTCTTCATGTGGGGC | CGGGTTCTTAAAGTTGCCTCCATT |
| P27 | PFF0165c | TCTCTTCTACATACGCTTTATTCA | GATAATTCGTTTAATGAGGAGTCCA |
| P35 | PF14_0045 | TCGTAGAAGAAGATGCGATGCT | TTGTGTCCACCTAAATATTCATAA |
| P38 | PF14_0089 | CGGAGGAACAATATGATAGTGATGATG | TCTTCTTCACTTAATTTTGACGAAAGGA |
| P77 | PF08_0048 | TGCAGACAAGACCATTCAAGTTAGGTATTG | ACCAGAAGGTAAATTCAGGAGTTTTGGCTGGTTC |
| P83 | PFC0345w | CACTCCTGTGTGCAGACTGTTTGT | CTTGTTCAGGAAGAAAGTGTTCGT |
| P90 | PFD0520c | GACGCCTCCGCCTGTATGAATAAT | CTTGAGCTTTAGAAATTCGTACTTTGTT |
| P97 | PFB0315w | ACTATACGTGAGTCATTCCGGGT | CGTAACATAGGTGAAGGAGAAGATG |
